# Apolipoprotein E polymorphism and the risk of intracranial aneurysms in a Chinese population

**DOI:** 10.1186/s12883-016-0537-z

**Published:** 2016-01-29

**Authors:** Hao Liu, Ping Mao, Changhou Xie, Wanfu Xie, Maode Wang, Haitao Jiang

**Affiliations:** Department of Neurosurgery, The First Affiliated Hospital of Xi’an Jiao Tong University, West Yanta Road No.277, Xi’an, 710061 China

**Keywords:** Apolipoprotein E, Gene polymorphism, Intracranial aneurysms

## Abstract

**Background:**

The relationship between the apolipoprotein E (*APOE*) polymorphism and intracranial aneurysms has previously only been studied in Russia and Japan but not in Chinese populations. The purpose of this study was to investigate the association between *APOE* polymorphism and the risk of intracranial aneurysms in a Chinese population.

**Methods:**

The study population consisted of 150 intracranial aneurysms patients and 150 matched control subjects. The *APOE* gene polymorphism was analyzed using polymerase chain reaction-restriction fragment length polymorphism (PCR-RFLP).

**Results:**

Patients with intracranial aneurysms had a significantly higher frequency of *APOE* E2/E2 genotype [odds ratio (OR) =9.51, 95 % confidence interval (CI) = 1.19, 76.04; *P* = 0.03] and *APOE* E2/E3 genotype (OR = 1.87, 95 % CI = 1.03, 3.40; *P* = 0.04) than healthy controls. The *APOE* E4/E4 genotype frequencies (OR = 0.09, 95 % CI = 0.01, 0.74; *P* = 0.03) in the intracranial aneurysms group were significantly lower than those in the controls group. When stratified by the site, shape, size and the Fisher Grade of intracranial aneurysms, no statistically significant result was observed.

**Conclusion:**

Our study suggested that *APOE* polymorphism might be associated with intracranial aneurysms in Chinese population. Additional studies are needed to confirm this finding.

## Background

Intracranial aneurysm is a fairly common condition that is often asymptomatic until the time of rupture. A systematic review of studies involving more than 56,000 patients found that unruptured intracranial aneurysms occur in 3.6 to 6 % of the general population [1]. Rupture of intracranial aneurysms accounts for more than 90 % of subarachnoid hemorrhage cases [2]. Intracranial aneurysms may result from diseases acquired during life, or from genetic conditions. Lifestyle diseases including hypertension, smoking, excess alcohol consumption, and obesity are associated with the development of aneurysms [3–5]. Genome-wide association studies (GWAS) of intracranial aneurysm in European and Japanese case-control cohorts have identified several new risk loci [6–9].

Apolipoprotein E (apoE), a 299-amino acid, arginine-rich glycoprotein, is an integral surface component of chylomicrons, very-low-density lipoproteins, and some subclasses of high-density lipoproteins [10, 11]. The *APOE* gene is polymorphic, consisting of 3 common alleles (E2, E3, and E4) and 6 different genotypes (E2/E2, E2/E3, E2/E4, E3/E3, E3/E4 and E4/E4) [11]. *APOE* gene polymorphisms seem to have some impact among patients with cardiovascular disease [12].

The relationship between the *APOE* polymorphism and intracranial aneurysms has previously only been studied in Russia and Japan but not in Chinese populations [13, 14]. The purpose of this study was to investigate the association between *APOE* polymorphisms and the risk of intracranial aneurysms in a Chinese population.

## Methods

### Study population

A hospital-based case-control study was conducted in 150 patients with intracranial aneurysms and 150 age- and gender-matched healthy controls during the years 2009 to 2014 in the Department of Neurosurgery, The First Affiliated Hospital of Xi’an Jiao Tong University, China. Peripheral blood specimens and demographic, medical, and family histories were obtained from sequentially ascertained, unrelated patients. Intracranial aneurysms in the cases were confirmed by magnetic resonance imaging (MRI) or computed tomography angiography (CTA) or cerebral angiography. Most intracranial aneurysms cases have no symptoms and may only be discovered during tests for another, usually unrelated, condition. The healthy control subjects were collected from the same geographic region and were attending a clinic for routine examination in the Department of Neurosurgery, The First Affiliated Hospital of Xi’an Jiao Tong University. The control subjects met the following criteria: (1) confirmation of the absence of intracranial aneurysms by digital subtraction angiography, magnetic resonance angiography, or 3-dimensional computed tomography angiography; (2) a similar age and sex distribution; (3) no medical history of any vascular disease, including intracranial aneurysms or subarachnoid hemorrhage; (4) no family history of intracranial aneurysms or subarachnoid hemorrhage in first-degree relatives; and (5) the same population as the cases. The Ethical Committee of the First Affiliated Hospital of Xi’an Jiao Tong University approved the study protocols, and all participants gave written informed consent according to the Declaration of Helsinki.

### DNA extraction and genotyping

The commercially available Qiagen kit (QIAGEN Inc., Valencia, CA, USA) was used to extract DNA from peripheral blood leukocytes. Polymerase chain reaction restriction fragment length polymorphism (PCR-RFLP) assay was applied to assess the *APOE* gene polymorphisms. Based on the GenBank reference sequence, the PCR primers were as follows: sense, ACAGAATTCGCCCCGGCCTGGTACAC; antisense, TAAGCTTGGCACGGCTGTCCAAGGA. The amplified PCR products were then digested with 2U of *Hha* I (New England BioLabs, Missisauga, ON, Canada) at 37 °C for 3 h. The resulting DNA fragments were electrophoresed on 3.5 % agarose gel and visualized under UV light after ethidum staining.

### Statistical analysis

The Statistical Analyses System (SAS) package (version 8.01; SAS Institute, Cary, NC) was used for statistical analysis. Differences between continuous variables were assessed by Student’s *t* test, while those between categorical variables were evaluated using Pearson *x*^2^ test. The existence of differences in genotypic frequencies between groups was assessed by means of Pearson *x*^2^ test and calculating the odds ratio (OR) with the 95 % confidence intervals (CI). A *P*-value was considered significant at a level of < 0.05. A chi-square test was used to confirm that the *APOE* genotype frequencies were in Hardy-Weinberg equilibrium.

## Results

The characteristics of patients with intracranial aneurysms and controls were shown in Table 1. As to clinical characteristics, there was no statistical significant difference in gender, age or the prevalence of risk factors including hypertension, smoking and drinking between the patient with intracranial aneurysms and control groups, as shown in Table 1. The site of aneurysm was as follows: anterior cerebral artery, 67 (44.7 %); internal carotid artery, 53 (35.3 %); and middle cerebral artery, 30 (20.0 %). The shape of aneurysm was as follows: saccular, 119 (79.3 %); fusiform, 24 (16.0 %); dissecting, 7 (4.7 %). The size of aneurysm was as follows: small (<15 mm), 106 (70.7 %); larger (15–25 mm), 37 (24.7 %); giant (>25 mm), 7 (4.6 %). The Fisher Grade was as follows: Grade 1, 14 (9.4 %); Grade 2, 74 (49.3 %); Grade 3, 44 (29.3 %); Grade 4, 18 (12.0 %) (Table 1).

**Table 1:** Characteristics of patients with intracranial aneurysms and controls

	Aneurysms	Controls	*P*
Total No.	150	150	
Gender (Male/Female)	67/83	65/85	0.82
Age (years)	46.7 ± 10.9	47.2 ± 11.2	0.70
Hypertension (Yes/No)	53/97	49/101	0.63
Smoking (Yes/No)	37/113	35/115	0.79
Drinking (Yes/No)	31/119	30/120	0.89
Site of aneurysm (%)			
ACA	67(44.7)	/	
ICA	53(35.3)	/	
MCA	30(20.0)	/	
Shape of aneurysm (%)			
Saccular	119(79.3)	/	
Fusiform	24(16.0)	/	
Dissecting	7(4.7)	/	
Size of aneurysm (%)			
Small (<15 mm)	106(70.7)	/	
Larger (15-25 mm)	37(24.7)	/	
Giant (>25 mm)	7(4. 6)	/	
The Fisher Grade			
Grade 1	14(9.4)	/	
Grade 2	74(49.3)	/	
Grade 3	44(29.3)	/	
Grade 4	18(12.0)	/	

ACA, anterior cerebral artery; ICA, internal carotid artery; MCA, middle cerebral artery. Smoking was defined as non-smoker (smoked less than 100 cigarettes in lifetime) and smoker. Drinking was defined as non-drinker and drinker (consumed more than one cup, 200 mL, per day)

Patients with intracranial aneurysms had a significantly higher frequency of *APOE* E2/E2 genotype (OR = 9.51, 95 % CI = 1.19, 76.04; *P* = 0.03) and *APOE* E2/E3 genotype (OR = 1.87, 95 % CI = 1.03, 3.40; *P* = 0.04) than healthy controls (Table 2). The *APOE* E4/E4 genotype frequencies (OR = 0.09, 95 % CI = 0.01, 0.74; *P* = 0.03) in the intracranial aneurysms group were significantly lower than those in the controls group (Table 2). When stratified by the site, shape, size and the Fisher Grade of intracranial aneurysms, no statistically significant result was observed (Table 3).

**Table 2:** Genotype frequencies of *APOE* gene polymorphisms among intracranial aneurysms cases and controls

Genotype	Aneurysms	Controls	OR (95 % CI)	*P* value
E2/E2	9(6.0)	1(0.6)	9.51(1.19,76.04)	0.03
E2/E3	35(23.3)	21(14.0)	1.87(1.03,3.40)	0.04
E2/E4	4(2.7)	7(4.7)	0.56(0.16,1.95)	0.36
E3/E3	76(50.7)	80(53.3)	0.90(0.57,1.41)	0.64
E3/E4	25(16.7)	31(20.7)	0.77(0.43,1.38)	0.38
E4/E4	1(0.6)	10(6.7)	0.09(0.01,0.74)	0.03

**Table 3:** Stratification analysis of *APOE* genotype frequency in intracranial aneurysms

Variable	Cases	E2/E2		E2/E3		E2/E4		E3/E3		E3/E4		E4/E4	
		n (%)	*P*	n (%)	*P*	n (%)	*P*	n (%)	*P*	n (%)	*P*	n (%)	*P*
Site of aneurysm (%)	150	9(6.0)		35(23.3)		4(2.7)		76(50.7)		25(16.7)		1(0.6)	
ACA	67	4(6.0)	0.99	16(23.9)	0.95	2(3.0)	0.90	33(49.2)	0.91	11(16.4)	0.97	1(1.5)	0.57
ICA	53	3(5.7)	0.93	12(22.6)	0.94	1(1.9)	0.76	28(52.8)	0.88	9(17.0)	0.97	0(0)	0.97
MCA	30	2(6.7)	0.90	7(23.3)	1.00	1(3.3)	0.84	15(50.0)	0.97	5(16.7)	1.00	0(0)	0.76
Shape of aneurysm (%)	150	9(6.0)		35(23.3)		4(2.7)		76(50.7)		25(16.7)		1(0.6)	
Saccular	119	5(4.2)	0.53	27(22.7)	0.92	2(1.7)	0.60	64(53.8)	0.78	20(16.8)	0.98	1(0.8)	0.87
Fusiform	24	3(12.5)	0.30	6(25.0)	0.89	1(4.1)	0.70	10(41.7)	0.63	4(16.7)	1.00	0(0)	0.66
Dissecting	7	1(14.3)	0.44	2(28.6)	0.81	1(14.3)	0.16	2(28.6)	0.48	1(14.3)	0.89	0(0)	0.26
Size of aneurysm (%)	150	9(6.0)		35(23.3)		4(2.7)		76(50.7)		25(16.7)		1(0.6)	
Small (<15 mm)	106	4(3.8)	0.45	26(24.5)	0.86	3(2.8)	0.94	54(50.9)	0.98	18(17.0)	0.96	1(1.0)	0.81
Larger (15–25 mm)	37	4(10.8)	0.35	7(18.9)	0.64	1(2.7)	0.99	19(51.4)	0.97	6(16.2)	0.96	0(0)	0.86
Giant (>25 mm)	7	1(14.3)	0.44	2(28.6)	0.81	0(0)	0.60	3(42.8)	0.81	1(14.3)	0.89	0(0)	0.26
The Fisher Grade	150	9(6.0)		35(23.3)		4(2.7)		76(50.7)		25(16.7)		1(0.6)	
Grade 1	14	1(7.1)	0.87	3(21.4)	0.90	0(0)	0.93	8(57.2)	0.80	2(14.3)	0.53	0(0)	0.45
Grade 2	74	5(6.8)	0.84	18(24.3)	0.90	2(2.7)	0.99	35(47.3)	0.78	13(17.6)	0.89	1(1.3)	0.62
Grade 3	44	2(4.5)	0.73	10(22.7)	0.95	2(4.5)	0.55	23(52.3)	0.92	7(15.9)	0.92	0(0)	0.94
Grade 4	18	1(5.5)	0.94	4(22.2)	0.93	0(0)	0.95	10(55.6)	0.83	3(16.7)	1.00	0(0)	0.55

## Discussion

Although lot of studies have been conducted to examine the association of genetic polymorphism and intracranial aneurysms, the relationship between the *APOE* polymorphism and intracranial aneurysms has previously only been studied in Russia and Japan but not in Chinese populations [13, 14]. Evidence from a case-control study suggested that the collagen type I alpha2 gene polymorphism was associated with intracranial aneurysms in a subset of the German population [15]. Authors of a case-control study suggested that the *IL-12A* and *IL-12B* independently and jointly was involved in the susceptibility to intracranial aneurysms in a Chinese population [16]. Evidence from a meta-analysis included six case-control studies, which included 1188 intracranial aneurysms cases and 4099 controls, suggested that *IL-6* promoter polymorphisms (-174G/C and -572G/C) were associated with intracranial aneurysms [17]. Authors of a case-control study in a Chinese population suggested that the *miR-34b/c* rs4938723CC and *TP53* Arg72-Pro polymorphisms may be involved in the susceptibility to intracranial aneurysms [18]. Evidence from a case-control study indicated that the elastin gene polymorphism might be associated with the formation and the rupture of intracranial aneurysms [19]. Evidence from a genetic meta-analysis of 8 genes and 13 polymorphisms in approximately 20,000 individuals showed that there was a likely genetic basis to sporadic intracranial aneurysms. However, the evidence base was small when compared against other complex disorders [20]. Authors of a case-control study suggested that the *NFKB1* -94ins/del ATTG polymorphism might contribute to the risk of intracranial aneurysms [21]. Polymorphisms within the kallikrein gene cluster are associated with intracranial aneurysms suggesting that the kallikreins are important candidate genes for intracranial aneurysms [22]. Evidence from a case-control study suggested that the angiotensin-converting enzyme DD genotype might be a protective factor for intracranial aneurysms in a Chinese population [23]. Evidence from a meta-analysis of case-control studies also suggested that there was a close relationship between angiotensin-converting enzyme I/D polymorphism and intracranial aneurysms risk [24]. The study by Pannu et al. and Peters et al. supported a role for *MMP-9* in the pathogenesis of intracranial aneurysms [25, 26]. Genome-wide association study for intracranial aneurysm in the Japanese population identified three candidate susceptible loci and a functional genetic variant at *EDNRA* [8].

The *APOE* gene polymorphisms were also associated with many other diseases. Authors of a case-control study showed that the *APOE* epsilon polymorphism has the expected impact on the plasma lipid profile and the rs4420638 G allele may counterbalance the deleterious effect of the E4 allele on low-density lipoproteins (LDL)-cholesterol levels in an Algerian population [11]. Evidence from a meta-analysis of 29 studies suggested that *APOE* gene polymorphisms was associated with the risk of vascular dementia [27]. Evidence from a meta-analysis of 28 case-control studies provided evidence for an association between the *APOE* E4 allele and frontotemporal lobar degeneration [28]. Authors of a case-control study found that *APOE* gene polymorphism was associated with maximal oxygen uptake levels after exercise training in Chinese young adult [29]. Evidence from a meta-analysis of experimental and human studies suggested an association between *APOE* gene expression and its gene polymorphism with nephrotic syndrome susceptibility [30]. Evidence from a meta-analysis of 45 studies found that *APOE* gene polymorphisms were associated with essential hypertension [31]. Authors of a hospital-based case-control study suggested that *APOE* E3/E4 genotype was associated with a higher lower extremity deep venous thrombosis risk [32]. Evidence from a meta-analysis of seven studies suggested that the *APOE* polymorphisms were associated with the risk of psoriasis, especially E2 and E3 alleles [33]. A meta-analysis of seven studies suggested an association between *APOE* E4 mutation and increased risk of recurrent pregnancy loss [34]. Evidence from a meta-analysis of 6977 subjects provides evidence that *APOE* E2 mutation is associated with multiple sclerosis risk [35]. Evidence from a meta-analysis included 29 studies suggested that *APOE* E4 allele was associated with an increased risk of developing cerebral infarction in Chinese population [36].

The exact molecular mechanism of the association between APOE polymorphism and the risk of intracranial aneurysms remains relatively unclear. APOE plays a critical role in redistributing lipids among central nervous system cells for normal lipid homeostasis [37, 38], repairing injured neurons [39], maintaining synaptodendritic connections [40], neurite outgrowth [41], synaptic plasticity [42], mitochondrial resistance to oxidative stress [43], and glucose use by neurons and glial cells [44, 45]. A case-control study in Russia found that intracranial aneurysms with hypertension were associated with the e2 allele and the e2/e3 APOE genotype [13]. This was consistent with our study.

There are some limitations to the present study that should be mentioned. First of all, although this study suggested that *APOE* polymorphism was associated with intracranial aneurysms, more biological background data are needed to explain our results. The current finding might involve gene-to-environment interactions, which were not explored in the present study. Second, the sample size of this study is relatively small, which may not have enough statistical power to explore the real association. Third, this is a hospital based case control study and selection bias cannot be avoided and therefore the subjects may not be representative of the general population within China. Finally, these results should be interpreted with caution because the population was only from China, which reduces the possibility of confounding from ethnicity, however does not permit extrapolation of the results to other ethnic groups.

## Conclusions

In conclusion, our study suggested that *APOE* polymorphism might be associated with intracranial aneurysms in Chinese population. Additional studies are needed to confirm this finding.
